# Detection and Genotyping of *Coxiella burnetii* and *Coxiella*-Like Bacteria in Horses in South Korea

**DOI:** 10.1371/journal.pone.0156710

**Published:** 2016-05-31

**Authors:** Min-Goo Seo, Seung-Hun Lee, Dorene VanBik, In-Ohk Ouh, Sun-Hee Yun, Eunsang Choi, Yong-Soo Park, Sang-Eun Lee, Jong Wan Kim, Gil-Jae Cho, Oh-Deog Kwon, Dongmi Kwak

**Affiliations:** 1 College of Veterinary Medicine, Kyungpook National University, Daegu, South Korea; 2 Animal and Plant Quarantine Agency, Gimcheon, South Korea; 3 Division of Veterinary Service Lab, Institute of Public Health & Environment, Incheon, South Korea; 4 Smile Equine Clinic, Busan, South Korea; 5 Dept. of Horse Industry, Korea National College of Agriculture and Fisheries, Jeonju, South Korea; 6 Division of Malaria & Parasitic Disease, Korea National Institute of Health, Cheongju, South Korea; 7 Cardiovascular Research Institute, Kyungpook National University, Daegu, South Korea; Texas A&M Health Science Center, UNITED STATES

## Abstract

*Coxiella burnetii* and *Coxiella*-like bacteria (CLB) are genetically and ecologically distinct despite some genetic similarities. Furthermore, CLB are exceptionally diverse and widespread in ticks, but rarely detected in domestic animals. Since *Coxiella* bacteria can be transmitted from infected horses by inhalation or by coming in contact with ticks during activities such as horseback riding, it is necessary to study their prevalence. To the best of our knowledge, this is the first large-scale nationwide investigation of the prevalence of *C*. *burnetii* and CLB among horses reared in South Korea. Of 816 blood samples collected between 2007 and 2013, 11 (1.3%) were identified as *C*. *burnetii* by ELISA, and six (0.7%) as CLB by 16S rRNA sequencing. While a sequence from Jeju Island was similar (97.9–100%) to those within clade B, five sequences obtained from the northern region were categorized into a new clade, indicating the sequence diversity of the genus *Coxiella*. Studies until date had detected CLB only in ticks; here, we describe their detection in mammals. Given their zoonotic potential, strategic monitoring and appropriate control programs for *Coxiella* species need to be established.

## Introduction

*Coxiella burnetii*, an obligate intracellular gram-negative bacterium, is a zoonotic pathogen that causes Q fever or coxiellosis. *C*. *burnetii* has been detected in species across the animal kingdom, including many domestic and wild mammals, birds, and arthropods such as ticks [1]. *C*. *burnetii* was originally named as *Rickettsia burnetii* on the basis of its similarity to *Rickettsia* species, but 16S rRNA analysis-based phylogeny placed this species in the genus *Coxiella*, which belongs to the gamma subdivision of the phylum Proteobacteria [2]. *C*. *burnetii* is currently the only species in this genus [3]; however, another species, *C*. *cheraxi*, presumed to belong to this genus, has been detected in crayfish [4]. Moreover, the fact that ticks transmit both *C*. *burnetii* and *Coxiella*-like bacteria (CLB) emphasizes the need to accurately discriminate between them [5]. In addition, questions about these bacteria remain, including the potential role of CLB in the population dynamics of ticks, and the possibility of CLB conversion leading to the emergence of Q fever [6].

Infertility and abortion caused by *C*. *burnetii* have been reported in numerous animals, but it is often difficult to identify the infection owing to its asymptomatic nature [7]. Stillbirth, abortion, and neonatal death caused by *C*. *burnetii* lead to economic loss in the horse industry [8]. To date, the role of horses as a reservoir for *C*. *burnetii* has not been extensively studied. *C*. *burnetii* is a well-known cause of abortion in ruminants; however, several recent studies have examined this characteristic in horses, too. *C*. *burnetii* DNA was detected in the aborted fetuses of horses, indicating the abortogenic nature of *C*. *burnetii* in horses [9,10]. *C*. *burnetii* DNA was also detected in the placenta of horses without any known abortion history in the Netherlands. Seven recent studies had detected *C*. *burnetii* in horse samples, in particular, in aborted fetuses, while another 34 studies determined the seroprevalence of *C*. *burnetii* in horses [7]. Therefore, horses should be considered as a reservoir for *C*. *burnetii* [11].

Although several studies have investigated *C*. *burnetii* infection in dairy cattle, goats, and water deer in South Korea [12–14], no studies have examined the occurrence of *C*. *burnetii* in horses. Recently, there has been a boom in the horse industry in South Korea, as the international trade of horses has increased. The potential risk of transmission of *Coxiella* species to humans may increase after exposure to infected horses or ticks during horseback riding. Therefore, the objective of this study was to detect and survey the current epidemiological prevalence and distribution of *C*. *burnetii* in horses reared in South Korea, by using ELISA and PCR.

## Materials and Methods

### Ethics statement

This study did not receive approval from the Institutional Animal Care and Use Committee (IACUC) at Kyungpook National University (KNU) in 2007, as the IACUC at KNU evaluates laboratory animals maintained in indoor facilities, not outdoor animals. Equine veterinarians collected blood samples at horse farms after receiving consent from the horse owners.

### Sample size determination and sample collection

The total number of horses reared in South Korea in 2014 was recorded at 25,819 [15]. The sample size for this study was determined using the following formula, with an expected disease prevalence of 50%, accepted absolute error of 5%, and a confidence level of 99% by using a simple random sampling design [16]:

$$n=\frac{1.96^{2}p_{exp}(1-p_{exp})}{d^{2}}$$

where *n* = required sample size, *p*_exp_ = expected prevalence, and *d* = desired absolute precision

**Fig 1:**
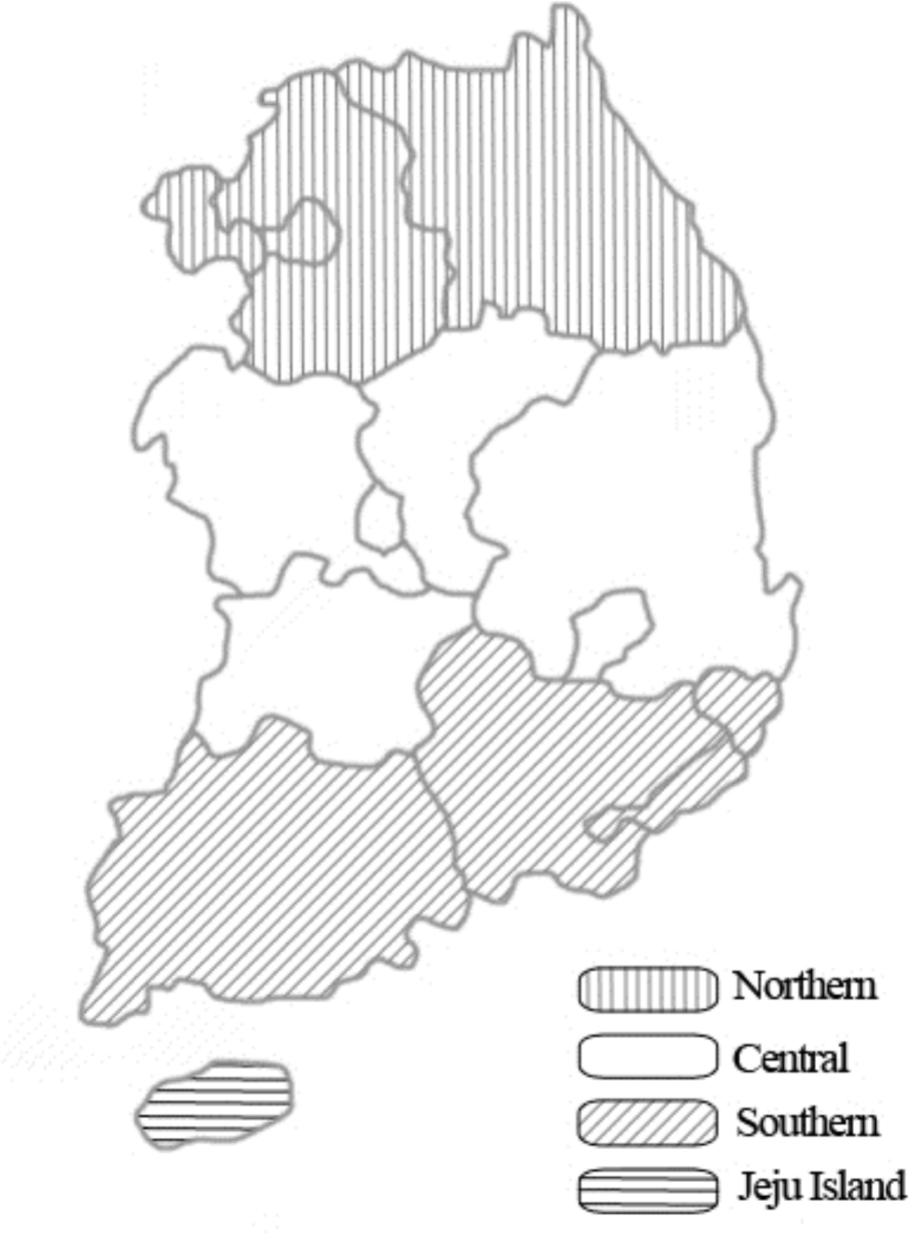
A map of South Korea showing the four different study regions where blood samples were collected from horses to detect *Coxiella* species.

According to the formula, a minimum of 664 samples was required. In this study, 816 horses were randomly selected from multiple regions in South Korea, between 2007 and 2013 (Fig 1). Following blood collection from the jugular vein, whole blood was used for PCR, and serum samples were used for serology. Age, sex, breed, and region were recorded for data analysis, and missing information was recorded as “unknown” (Table 1). The mean age of the study animals was 7.2 years, with a standard deviation of 4.6 years.

**Table 1:** Detection of *Coxiella* infection among horses raised in Korea between 2007 and 2013.

Group		No. tested	No. (%) of horses			
			ELISA		PCR	
			Positive	95% CI^†^	Positive	95% CI^†^
**Region**	**Northern**	295	7 (2.4)	0.6–4.1	5 (1.7)	0.2–3.2
	**Central**	184	2 (1.1)	0–2.6	0	0
	**Southern**	243	2 (0.8)	0–2.0	0	0
	**Jeju Island**	94	0	0	1 (1.1)	0–3.1
**Breed**	**Thoroughbred**	566	11 (1.9)	0.8–3.1	5 (0.9)	0.1–1.7
	**Native Korean pony**	109	0	0	1 (0.9)	0–2.7
	**Warm blood**	61	0	0	0	0
	**Mixed**	80	0	0	0	0
**Sex**	**Male**	159	3 (1.9)	0–4.0	1 (0.6)	0–1.9
	**Female**	283	5 (1.8)	0.2–3.3	3 (1.1)	0–2.3
	**Castrated**	280	3 (1.1)	0–2.3	1 (0.4)	0–1.1
	**Unknown**	94	0	0	1 (1.1)	0–3.1
**Age**	**<5**	271	5 (1.8)	0.2–3.5	5 (1.8)*	0.2–3.5
	**5–10**	249	4 (1.6)	0.1–3.2	0	0
	**>10**	202	2 (1)	0–2.4	0	0
	**Unknown**	94	0	0	1 (1.1)	0–3.1
**Total**		816	11 (1.3)	0.6–2.1	6 (0.7)	0.2–1.3

*Significantly different, *p* < 0.05

^†^CI = confidence interval.

### Serology

Serum samples were checked for the presence of antibodies against *C*. *burnetii* by ELISA with the ID Screen Q Fever Indirect Multi-species Kit (IDvet, Montpellier, France), in accordance with the manufacturer’s instructions. The microwells were coated with *C*. *burnetii* phases I and II. The optical density ratio of the sample and the positive control (S/P) was calculated for each sample as follows:

$$\text{Value}\;(\%)=\frac{{OD}_{sample}-{OD}_{negative\;control}}{{OD}_{positive\;control}-{OD}_{negative\;control}}\times 100$$

Samples with an S/P value greater than 50% were considered positive; values between 40% and 50% were deemed doubtful, and those less than 40% were determined to be negative. Doubtful results were considered negative.

### DNA extraction and PCR

Genomic DNA was extracted from whole blood, using the commercial DNeasy Blood and Tissue Kit (Qiagen, Melbourne, Australia) according to the manufacturer’s instructions. The extracted DNA was stored at −20°C until use. The commercial AccuPower HotStart PCR Premix Kit (Bioneer, Daejeon, South Korea) was used for PCR amplification. Multiple primer sets were used to amplify 16S rRNA of the genus *Coxiella*. *Coxiella* was first screened by nested PCR (nPCR), as previously described [6,17]. First-round PCR was performed with the primers Cox16SF1 (5′-CGTAGGAATCTACCTTRTAGWGG-3′) and Cox16SR2 (5′-GCCTACCCGCTTCTGGTACAATT-3′), which produced amplicons with 1,321–1,429 bp. Then, nPCR was performed using the primers Cox16SF2 (5′-TGAGAACTAGCTGTTGGRRAGT-3′) and Cox16SR2, which produced amplicons with 624–627 bp. Samples yielding amplicons of the expected size were sequenced using the primers Cox16SF1 and Cox16SR1 (5′-ACTYYCCAACAGCTAGTTCTCA-3′), which produced amplicons with 719–826 bp. All PCR amplifications were performed using the Mastercycler Pro (Eppendorf, Hamburg, Germany), with a pre-denaturation cycle at 93°C for 3 min, followed by 30 cycles of denaturation at 93°C for 30 s, annealing at 56°C for 30 s, and polymerization at 72°C for 1 min, with a final post-polymerization cycle at 72°C for 5 min. PCR products of the second round of amplification were evaluated by electrophoresis, using 10 μl of the reaction mixture and a 100 bp DNA ladder (Bioneer) in 1.5% agarose gel for 30 min at 100 V, and visualized using UV transillumination, after ethidium bromide staining.

### DNA sequencing and phylogenetic analysis

Purified amplicons, obtained from nPCR using the primers Cox16SF1 and Cox16SR1, were sent to Solgent (Daejeon, South Korea) for nucleotide sequencing. The sequences were analyzed using the multiple sequence alignment program CLUSTAL Omega (ver. 1.2.1). Alignment results were corrected using BioEdit (ver. 7.2.5). Phylogenetic analysis was performed using MEGA (ver. 6.0) and the aligned sequences of *Coxiella* 16S rRNA were compared to determine homology. Stability of the trees obtained was estimated by bootstrap analysis with 1,000 replicates.

### Statistical analysis

Chi-square test was used to analyze significant differences among the groups. Data for the “unknown” group were disregarded in the chi-square test. A *p* value of < 0.05 was considered statistically significant. The analytical software package GraphPad Prism version 5.04 (GraphPad Software Inc., La Jolla, CA, USA) was used for statistical analysis. A confidence interval (CI) of 95% was calculated for all estimates.

## Results

### Serological and molecular analyses

As shown in Table 1, the sera of 11 horses (1.3%, 95% CI: 0.6–2.1) tested positive for *C*. *burnetii* by ELISA. In addition, the sera of six horses (0.7%, 95% CI: 0.2–1.3) tested positive for CLB by 16S rRNA sequencing. With respect to region, sex, and breed, no statistically significant differences were observed. However, prevalence was relatively high (2.4% by ELISA and 1.7% by PCR) in the northern region compared to other regions. In Jeju Island, none of the 94 tested samples were positive for *C*. *burnetii*, but one (1.1%, 95% CI: 0–3.1), a native Korean pony, tested positive for CLB by PCR. Although some thoroughbreds were positive by either ELISA or PCR, none tested positive by both assays. When PCR data were analyzed by age, prevalence was observed to be significantly higher (*p* < 0.05) in horses less than 5 years of age (1.8%, 95% CI: 0.2–3.5).

### Prevalence based on ELISA and PCR data

None of the samples tested positive by both assays. Six (0.7%) samples were PCR+/ELISA−, 11 (1.3%) samples were PCR−/ELISA+, and 799 (97.9%) samples were PCR−/ELISA− (Table 2).

**Table 2:** Comparison of *Coxiella* detection by ELISA and PCR.

		PCR		Total
		No. positive	No. negative	
**ELISA**	**No. positive**	0	11	11
	**No. negative**	6	799	805
**Total**		6	810	816

### DNA sequencing and phylogenetic analysis

**Fig 2:**
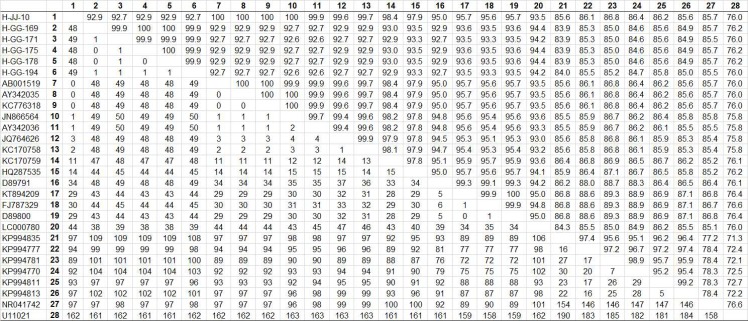
Comparison of *Coxiella* 16S rRNA nucleotide sequences. The upper matrix shows percent identity between the partial sequences of the *Coxiella* 16S rRNA gene. The lower matrix presents the number of differences in nucleotide bases.

Among the six samples producing an amplicon of the expected size from *Coxiella* 16S rRNA, one sequence (H-JJ-10) from Jeju Island and five sequences (H-GG-169, 171, 175, 178, and 194) from the northern region of South Korea were studied. This analysis revealed that these sequences shared 92.7–100% similarity. The sequences of *Coxiella* 16S rRNA obtained from six horses were deposited in GenBank (accession nos. KT835658–KT835661, KU324470–KU324471). Comparative analysis of the 16S rRNA nucleotide sequences from the Korean samples with the 22 *Coxiella* isolates included in the GenBank database is shown in Fig 2.

**Fig 3:**
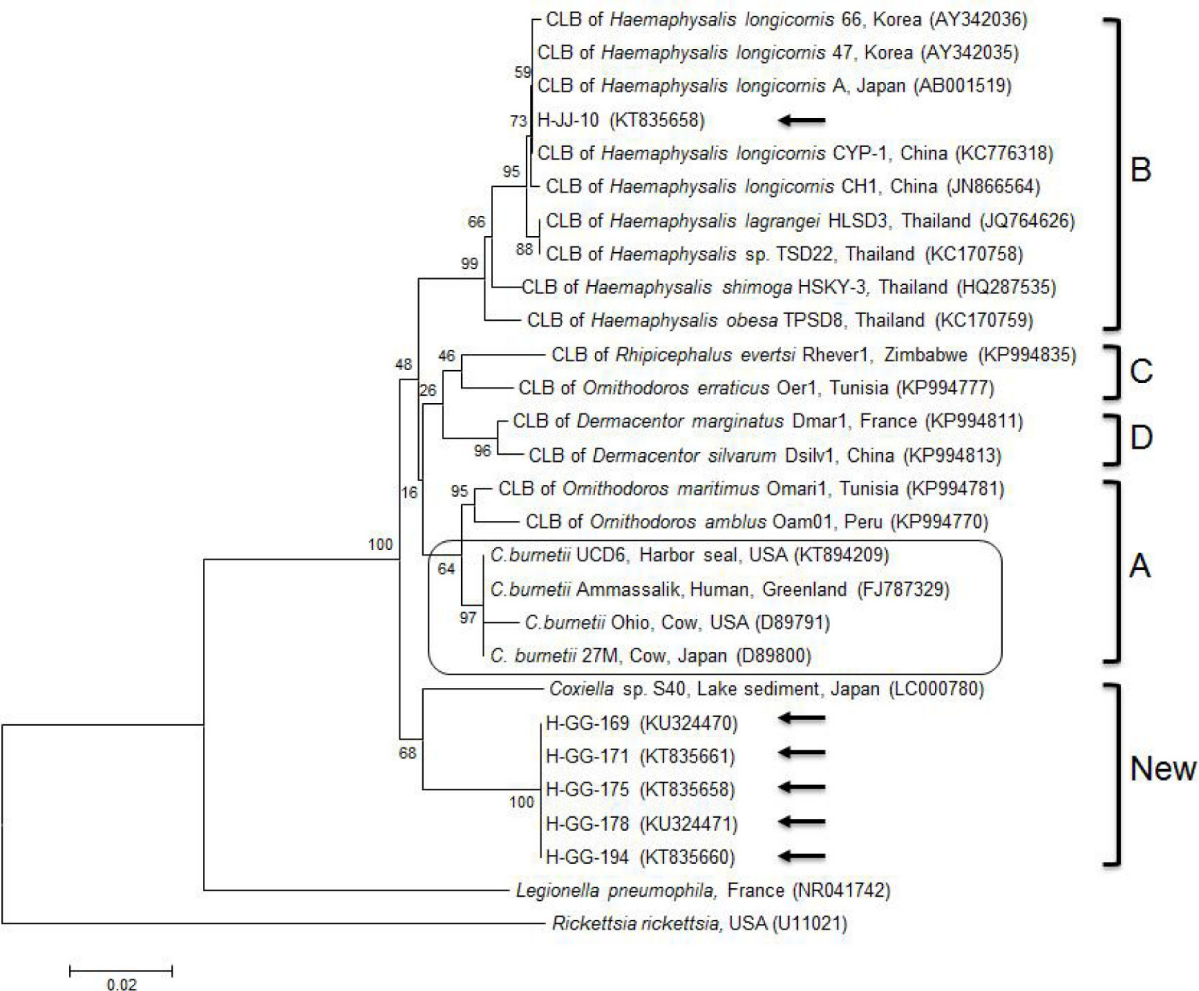
Phylogenetic tree constructed using Kimura/neighbor-joining methods based on 16S rRNA sequences of *Coxiella*. The four clades (A–D) of *Coxiella* are categorized. The sequences of *Coxiella*-like bacteria obtained in this study are marked using arrows. The *C*. *burnetii* group is found within clade A. The accession numbers of sequences obtained from GenBank are shown with the sequence names and countries of origin. Numbers on the branches indicate bootstrap support (1,000 replicates). Scale bar indicates a phylogenetic distance of 0.02 nucleotide substitution per position. CLB = *Coxiella*-like-bacteria.

*Coxiella* 16S rRNA sequences were compared to published sequences (available in GenBank). This phylogenetic analysis (Kimura/neighbor joining) showed that one sequence (H-JJ-10) from Jeju Island shared high similarity (97.9–100%) with those of nine CLB strains, within clade B, isolated from *Haemaphysalis* ticks, in South Korea (AY342035, AY342036), Japan (AB001519), China (KC776318, JN866564), and Thailand (JQ764626, KC170758, KC170759, and HQ287535) (Figs 2 and 3). In contrast, five sequences (H-GG-169, 171, 175, 178, and 194) from the northern region were clustered into a new clade, showing 94.2–94.4% similarity to *Coxiella* sp. S40 from Japan (LC000780), again within the same new clade (Figs 2 and 3).

## Discussion

In 1997, the development of 16S rRNA sequencing led to the first instance of identification of CLB in three species of ticks [18]. The sequences of CLB 16S rRNA were closely related to those of *C*. *burnetii*, which indicated diversity within the genus *Coxiella*, a previously overlooked aspect [19]. *C*. *burnetii* and CLB are genetically and ecologically distinct, despite genetic similarities. Furthermore, CLB are exceptionally diverse and widespread in ticks, but rarely described in domestic animals; however, CLB have recently been reported to be a leading cause of lethal systematic infections in domestic birds [20–22]. There is an important risk of misidentification, given that the current protocols for detecting *C*. *burnetii* in ticks depend on PCR-based detection of a single gene, without subsequent confirmation by sequencing [23].

An increase in the seroprevalence of *C*. *burnetii* in ruminant herds is a useful index for studying their occurrence in humans [24]. This could also be extrapolated to *C*. *burnetii* infections in horses. The primary route of transmission to humans is via nasal inhalation, and the rate of tick-borne transmission of Q fever in humans is considered low. However, some cases of possible tick-borne transmission have been reported [25, 26]. Those studies described patients with serological and clinical evidence of a tick-borne disease and subsequent or concomitant Q fever after tick bites. Furthermore, a case report indicates that *C*. *burnetii* could be spread by various ticks during horseback riding [27]. This might be a different approach of transmission involving horses. Our study confirms the results of a previous study indicating that humans can be exposed to *C*. *burnetii* from infected horses through contact with ticks during horseback riding; contaminated stable materials could also expose humans to polluted air or dust from the environment. In South Korea, the popularity of horseback riding has surpassed that of horseracing.

Recently, a meta-analysis of studies performed over an extended period across countries showed that the mean seroprevalence of *C*. *burnetii* in horses was expected to be 15.8% (95% CI: 9.6–23%) [7]. However, seroprevalence rates differ with the geographical area, test design, population, cut-off value used, year, diagnostic method, and sensitivity and specificity of the assay [7]. In the present study, the prevalence of *C*. *burnetii* was found to be 1.3% by ELISA and 0.7% by PCR among the 816 horses tested. Although we initially expected to detect *C*. *burnetii* in horses by PCR, the sequencing data indicate that only CLB were detected in horses. Because CLB are known to exist in the salivary glands of ticks, they could be transmitted to humans and vertebrates during blood sucking. Potential tick-to-vertebrate transmission of CLB is likely because ticks occur worldwide and feed on various hosts [5]. Because CLB detection has been restricted to ticks, CLB might pose a much lesser threat to vertebrates than *C*. *burnetii* [23]. However, the risk of vertebrate infection by CLB is unknown, as these bacteria have not been detected in vertebrates or associated with clinical symptoms [6]. Thus, to the best of our knowledge, the present study is the first to report the detection of CLB in a mammalian species, namely, the horse. Further studies should be performed to determine whether CLB infection manifests the clinical signs of the disease in mammals, including humans.

This study found relatively low positive yield of *Coxiella*, compared to other studies on ruminants in South Korea. A previous study using ELISA showed a detection rate of 24.2% (119/492) in dairy cattle and 54% (175/324) in bulk milk tanks in the southern regions [12]. Other studies also showed high infection rates in native Korean goats by ELISA (19.1%; 114/597) and PCR (9.5%; 57/597) in the central and southern regions [13], and wild Korean water deer by ELISA (9.2%; 18/196) and real-time PCR (6.6%; 13/196) in the northern, central, and southern regions [14]. Horse studies performed in other countries showed relatively low positive rates in aborted fetuses (0%; 0/122) by using PCR in Italy [7], blood (0%; 0/105) by complement fixation test in Denmark [28], aborted fetuses (1.5%; 6/407) by real-time PCR in France [10], and aborted fetuses (4.3%; 1/23) by real-time PCR in Germany [9]. However, much higher positivity was reported in aborted pregnancies (42.2%; 19/45) by PCR in Croatia [29], blood (22.2%; 4/18) by loop-mediated isothermal amplification in China [30], aborted or non-aborted placenta (7.7%; 3/39) by real-time PCR in the Netherlands [11], and blood (12.5%; 14/112) and urine (7.1%; 1/14) by real-time PCR in Australia [31].

The health status of horses that yielded positive results for *C*. *burnetii* could provide better insight into the pathogenesis of *C*. *burnetii*; however, this was not recorded in this study. While the data indicated no regional variations, the northern region did have a higher prevalence, as only one horse from Jeju Island was determined to be positive by PCR. This result was unexpected, as infectivity is likely to be observed in regions with a warm and wet climate, such as Jeju Island. However, the one native Korean pony infected with CLB was raised on Jeju Island. All horses of Jeju Island used in this experiment were native Korean ponies. Therefore, additional investigation is required for other breeds of horses reared on Jeju Island. The recent climate change has probably contributed to the widespread distribution of ticks [32] and an increase in the period of activity. With respect to breed, prevalence was mostly detected in thoroughbred horses, except one native Korean pony. Higher prevalence of *Coxiella* in thoroughbreds was expected based on the geographical distribution of thoroughbreds (46.7%, 12,066 horses were raised in South Korea) [15]. No significant difference was observed with respect to sex. Younger horses (< 5 years) showed significantly higher prevalence of CLB by PCR. This result was also unexpected because older animals are likely to have had more opportunities for exposure than younger animals [12,13].

In this study, 11 (1.3%) and six (0.7%) of the 816 horse blood samples tested positive for *Coxiella* by ELISA and PCR, respectively. None of the samples tested positive by both assays. The PCR assay employed detects genomic DNA common to all *Coxiella* species, including *C*. *burnetii* and CLB, while the ELISA is based on the specific detection of serum antibodies against only *C*. *burnetii*. Hence, one plausible explanation for the lack of consistency observed is that only CLB, but not *C*. *burnetii*, were present in the PCR-positive samples. Another possible explanation for this inconsistency may be the detection limit of ELISA, and the fact that this assay can detect antibodies from both active and prior infections. PCR can solely detect active infections [33]. The ID screen ELISA kit is adapted to mammalian IgG antibodies, and it was originally developed to react with cattle, goats, and sheep. However, it has also been used to react with antibodies against *C*. *burnetii* in blood samples from other mammalian species, including cats, foxes, and rodents [34].

Genotypically and phenotypically different features of CLB and *C*. *burnetii* could also conceivably lead to different rates of positivity. The widespread genetic variability in CLB strains compared to *C*. *burnetii* strains has led to a clear sub-classification of this genus into four largely divergent clades (A–D) [5]. The clustering of all *C*. *burnetii* strains within clade A indicates that the progenitor of *C*. *burnetii* was a tick-related bacterium that succeeded in infecting vertebrates. Based on the phylogenetic analysis, H-JJ-10 on Jeju Island is closely related to the CLB in *Haemaphysalis* ticks belonging to clade B. Therefore, further studies on CLB in ticks associated with horses are required, especially on Jeju Island. The H-JJ-10 clustered together with the CLB in *Haemaphysalis* ticks from South Korea, China, Japan, and Thailand, which implies a close epidemiological connection between these isolates. The five isolates from the northern region were placed into a new distinct clade. Because of the geographical differences between the northern region of the mainland and Jeju Island, the origin of *Coxiella* may differ, contributing to the diversity of this species in South Korea. Further molecular studies are needed to fully understand the diversity of the genus *Coxiella*.

To the best of our knowledge, this is the first report of a large-scale nationwide study to report the serological and molecular detection of the genus *Coxiella* in mammals, namely, horses. Nevertheless, further studies will be required to describe these CLB isolates, characterize their genetic relationship, and evaluate their potential to cause infections in vertebrates [5]. Considering the zoonotic potential of the genus *Coxiella* and climate change, which affects the widespread distribution of ticks and increases their period of activity, it is necessary to establish strategic monitoring, epidemiological insight, and appropriate control programs for *Coxiella* and other tick-borne diseases. Future research on possible cross-reactivity between *C*. *burnetii* and CLB will be essential to better evaluate the specificity of diagnostic assays and screening tools now used in vertebrates [5]. In addition, further epidemiological studies on *Coxiella* species in healthy horse herds, epizootics in horses, and positive cases of horse abortion are significant for better understanding *C*. *burnetii* or CLB infections.

## Conclusions

*C*. *burnetii* can be transmitted to humans following exposure to ticks during horseback riding. Global warming is expected to cause an increase in the number and distribution of ticks. To the best of our knowledge, this is the first large-scale nationwide investigation to study the prevalence of *C*. *burnetii* and CLB among 816 horses in South Korea. Eleven (1.3%) and six (0.7%) samples tested positive by ELISA and PCR, respectively. This study describes the first instance of CLB detection (previously restricted to ticks) in mammals, thereby providing evidence about the potential for transmission of Q fever-causing bacteria to humans from infected horses during activities such as horseback riding.
